# Analysis of benzo[*a*]pyrene metabolites formed by rat hepatic microsomes using high pressure liquid chromatography: optimization of the method

**DOI:** 10.2478/v10102-009-0024-0

**Published:** 2009-12-28

**Authors:** Michaela Moserová, Věra Kotrbová, Dagmar Aimová, Miroslav Šulc, Eva Frei, Marie Stiborová

**Affiliations:** 1 Department of Biochemistry, Faculty of Science, Charles University, Albertov 2030, 128 40 Prague 2, Czech Republic; 2 Department of Molecular Toxicology, German Cancer Research Center, 69 120 Heidelberg, Germany

**Keywords:** benzo[*a*]pyrene, metabolism, HPLC

## Abstract

A simple and sensitive method was developed to separate the carcinogenic polycyclic aromatic hydrocarbon (PAH), benzo[*a*]pyrene (BaP), and six of its oxidation metabolites generated by rat hepatic microsomes enriched with cytochrome P450 (CYP) 1A1, by high pressure liquid chromatography (HPLC). The HPLC method, using an acetonitrile/water gradient as mobile phase and UV detection, provided appropriate separation and detection of both mono- and di-hydroxylated metabolites of BaP as well as BaP diones formed by rat hepatic microsomes and the parental BaP. In this enzymatic system, 3-hydroxy BaP, 9-hydroxy BaP, BaP-4,5-dihydrodiol, BaP-7,8-dihydrodiol, BaP-9,10-dihydrodiol and BaP-dione were generated. Among them the mono-hydroxylated BaP metabolite, 3-hydroxy BaP followed by di-hydroxylated BaP products, BaP-7,8-dihydrodiol and BaP-9,10-dihydrodiol, predominated, while BaP-dione was a minor metabolite. This HPLC method will be useful for further defining the roles of the CYP1A1 enzyme with both *in vitro* and *in vivo* models in understanding its real role in activation and detoxification of BaP.

## Introduction

**Figure 1:**
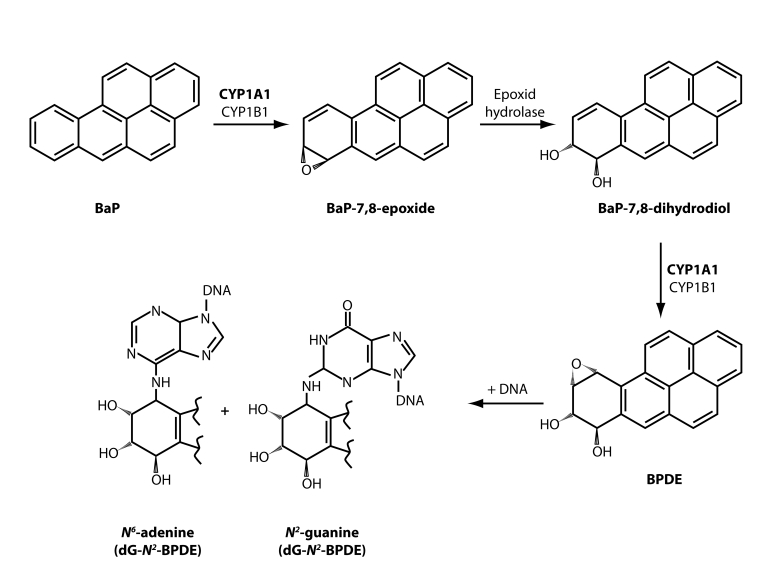
Metabolic activation and DNA adduct formation by benzo[*a*]pyrene. The typical three- step activation process with contribution of CYP1A1 or CYP1B1 and epoxide hydrolase leads to the formation of the ultimately reactive species, benzo[*a*]pyrene-7,8- dihydrodiol-9,10-epoxide (BPDE) that can react with DNA, forming adducts preferentially at guanine residues.

Benzo[*a*]pyrene (BaP) is the prototype compound of polycyclic aromatic hydrocarbons (PAH). BaP and other PAHs are produced mainly by incomplete combustion or pyrolysis of organic matter and are ubiquitous in the environment, leading to measurable background levels of exposure in the general population (IARC, 1983). Beside the inhalation of polluted air, the main routes of exposure are through tobacco smoke, diet (Phillips, 1999; 2002) and occupational exposition throughout, e.g. coal, coke or coal tar processing and use of coal tar products (IARC, 1983). BaP has been shown to cause cytotoxic, teratogenic, genotoxic, mutagenic, and carcinogenic effects in various tissues and cell types in organisms (Nebert, 1989; Ellard *et al*., 1991). Chronic exposure of laboratory animals to BaP has been associated with developments of cancer, primarily in the skin, stomach, and lungs as target tissues (IARC, 1983). BaP requires metabolic activation (Figure 1) prior to reaction with DNA, which is an essential step in the mechanism, by which BaP exerts its genotoxic effects.

Cytochrome P450 (CYP) enzymes in a combination with epoxide hydrolase are the major enzymes activating BaP to species binding to DNA. First, the CYP enzymes oxidize BaP to form an epoxide that is additionally converted to a dihydrodiol by epoxide hydrolase (Baird *et al*., 2005; Luch and Baird, 2005) (Figure 1). Further bioactivation step catalyzed by CYPs leads to the formation of the reactive species, benzo[a]pyrene-7,8-dihydrodiol-9,10-epoxide (BPDE) that can react with DNA, forming adducts preferentially at guanine residues. The 10-(deoxyguanosin-*N* ^2^-yl)-7,8,9-trihydroxy-7,8,9,10-tetrahydrobenzo[a]pyrene (dG-*N* ^2^-BPDE) adduct is the major adduct formed from BPDE in DNA *in vitro* and *in vivo* (Phillips, 2005) (Figure 1). Among CYP enzymes, CYP1A1 and 1B1 are widely accepted as the most important enzymes in such BaP metabolic activation (Baird *et al*., 2005; Luch and Baird, 2005). Nevertheless, controversial results have been found recently in several laboratories, showing a more important role of CYP1A1 *in vivo* in BaP detoxification than in its activation (Uno *et al*., 2004; 2006; Arlt *et al*., 2008). In order to explain such findings, activation and detoxification metabolism of BaP *in vitro* and *in vivo* should, therefore, be carefully re-evaluated. For such studies, appropriate methods separating and quantifying all BaP metabolites generated by CYPs in combination with epoxide hydrolase, besides methods evaluating the DNA adduct formation by this carcinogen, are necessary.

A variety of high pressure liquid chromatography (HPLC) procedures [reverse phase (RP)-HPLC] separating BaP metabolites was described by several authors for the last several decades (Selkirk *et al*., 1974; Selkirk, 1977; Angener et al., 1997; Gündel and Angerer, 2000; Hecht, 2001; Sasaki *et al*., 2002; Toriba *et al*., 2003; Wang *et al*., 2003; Sagredo *et al*., 2006; Jiang *et al*., 2007; Zhu *et al*., 2008). In the 1970′s, HPLC was used for separating BaP metabolites combined with UV or scintillation counter detection. However, UV detection in these studies was not sufficient to detect all BaP metabolites (Selkirk, 1977). Therefore, quantitation by measuring fluorescence as detection method was developed (Krahn *et al*., 1984). However, BaP diones, do not exhibit fluorescence, and the method does not quantitate individual metabolites prohibiting application of fluorescence detection for a detailed study of BaP metabolism. Thereafter, on-line or off-line radioactivity detection played a major role in the study of BaP metabolism (for summary see Zhu *et al*., 2008). The disadvantages of the radioactive method include cost, radiolabeled individual metabolites are not available, and it cannot be used for the analysis of environmental samples. The improvement of chromatographic separation methods have allowed for significantly better separation of BaP metabolites during the 1990′s (James *et al*., 1995; 1997; Kim *et al*., 1998). From 2000, HPLC coupled with mass spectrometry was introduced in the study of BaP metabolism, though there were still problems associated with high detection limits for BaP diones (van Schanke *et al*., 2001). Recently, ultra-performance liquid chromatography has been introduced with improved performance over traditional HPLC (Zhu *et al*., 2008). However, there are still several disadvantages in all these methods, predominantly cost of analyses, which prohibit them to be suitable for general use in most of laboratories. Therefore, the aim of this study was to improve the HPLC procedure to be effective for separation of BaP metabolites generated by BaP oxidation with rat hepatic microsomes and sensitive enough to be able to detect BaP metabolites with UV detection.

## Materials and methods

### Chemicals

Chemicals were obtained from the following sources: methanol (MetOH; HPLC supergradient) from Lachner (Czech Republic); acetonitrile (HPLC grade) from Merck (Darmstadt, Germany); benzo[a]pyrene (≥ 96% based on HPLC), NADP^+^, glucose-6-phosphate, phenacetine and bicinchoninic acid (2,2′-biquinoline-4,4′-dicarboxylic acid) from Sigma Chemical Co. (St. Louis, MO, USA) and glucose-6-phosphate dehydrogenase from Serva (Heidelberg, Germany). All these and other chemicals used in the experiments were of analytical purity or better.

### Preparation and characterization of microsomes

The animal experiment was conducted in accordance with the Regulations for the Care and Use of Laboratory Animals (311/1997, Ministry of Agriculture, Czech Republic), which is in compliance with the Declaration of Helsinki. Microsomes from rats pretreated with Sudan I were isolated from pooled livers of ten rats as described previously (Stiborová *et al*., 2003). Protein concentration in the microsomal fraction was measured using bicinchoninic acid protein assay (Wiechelman *et al*., 1988) with bovine serum albumin as a standard. The content of CYPs was determined by differential spectroscopy based on utilizing a characteristic absorption of the complex of this hemthiolate protein in reduced state with carbon oxide at 450 nm (Omura and Sato, 1964).

### Incubations

Incubation mixtures used for studying BaP metabolism contained 100 mM sodium phosphate buffer (pH 7.4), NADPH-generating system (1 mM NADP^+^, 10 mM D-glucose-6-phosphate, 1 U/ml D-glucose-6-phosphate dehydrogenase), 0.5 mg of microsomal protein, 50 µM BaP (dissolved in 5 µl methanol) in a final volume of 500 µl. The reaction was initiated by adding 50 µl of the NADPH-generating system. Control incubations were carried out either without enzymatic system (microsomes) or without NADPH-generating system or without BaP. After incubation in open tubes (37°C, 20 min), 5 µl of 1 mM phenacetine in methanol was added as an internal standard. BaP metabolites were extracted twice with ethyl acetate (2 × 1 ml) and evaporated to dryness. The samples were dissolved in 25 µl methanol and BaP metabolites formed in this system separated by HPLC.

### HPLC instrument used for HPLC analysis of BaP metabolites

HPLC analyses of BaP metabolites were performed using a Dionex system consisting of a Dionex pump P580, a UV/VIS Detector UVD 170S/340S, an ASI-100 Automated Sample Injector, a termobox COLUMN OVEN LCO 101 and an In-Line Mobile Phase Degasser Degasys DG-1210 Dionex controlled with Chromeleon™ 6.11 build 490 software. Chromatographic separation was performed on two types of reversed phase columns, Ultrasphere^®^ ODS, C18, 5 µm, 250 × 4.6 mm (Beckman-Coulter, USA) and Nucleosil^®^ 100-5 C18, 5 µm, 250 × 4 mm (Macherey Nagel, Germany).

### Chromatographic conditions

**Figure 2:**
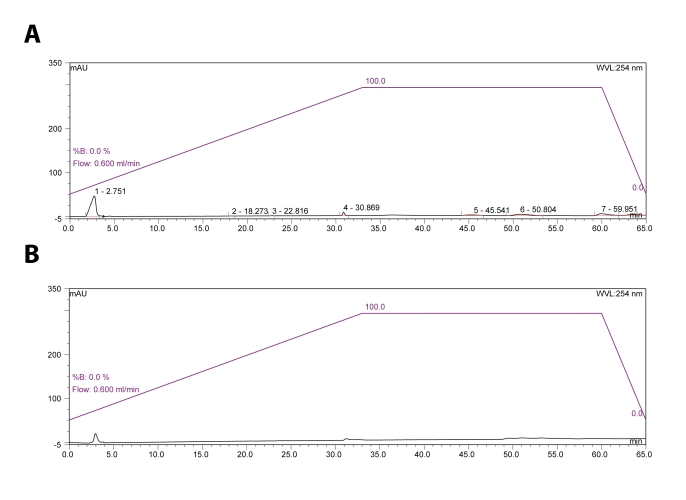
HPLC separation of the BaP metabolites generated by rat hepatic microsomes using Nucleosil^®^ C18 (**A**) and Ultrasphere^®^ C18 reverse phase columns (**B**). A linear gradient elution from 30% methanol to 70% methanol in 65 min. Flow rate 0.6 ml/min, UV detection at 254 nm.

Four different conditions to separate BaP metabolites were used. The first one was analogous to that used by Selkirk *et al*., 1974. Mobile phase A: 30% methanol (30:70 methanol: water, v/v), mobile phase B: 70% methanol (70:30 methanol: water, v/v), flow rate: 0.6 ml/min at operation temperature of 35°C, detection at 254 nm. The 20 µl sample was injected for HPLC analysis. BaP metabolite separation was performed using a Nucleosil^®^ C18 reverse phase column, (250 × 4 mm, 5 µm; Macherey Nagel). Linear gradient system started from 30% methanol to 70% methanol in 33 min and followed by isocratic elution of mobile phase B in 32 min (Procedure 1, Figure 2A).

In the second procedure (Procedure 2) the same conditions as in Procedure 1 were used except that separation of BaP metabolites was performed using an Ultrasphere^®^, ODS, C18 reverse phase column (250 × 4.6 mm, 5 µm) (Figure 2B).

In the Procedure 3 the experimental conditions were as follows: mobile phase: 85% acetonitrile (85:15 acetonitrile: water, v/v), flow rate: 0.6 ml/min at operation temperature of 35°C, detection at 254 nm. The 20 µl sample was injected for HPLC analysis.

**Figure 3:**
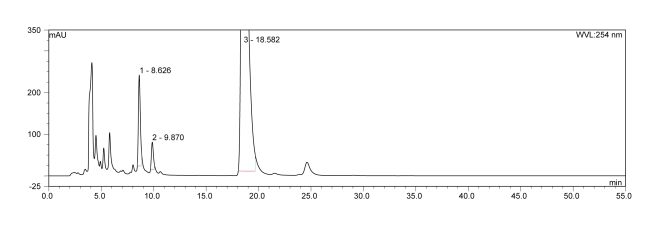
HPLC separation of the BaP metabolites generated by rat hepatic microsomes using a Nucleosil^®^ C18 reverse phase column. An isocratic elution of 85% acetonitrile in 55 min. Flow rate 0.6 ml/min, UV detection at 254 nm.

BaP metabolite separation was performed using the isocratic elution of mobile phase in 55 min, on a Nucleosil^®^ C18 reverse phase column, (250 × 4 mm, 5 µm; Macherey Nagel) (Figure 3).

**Figure 4:**
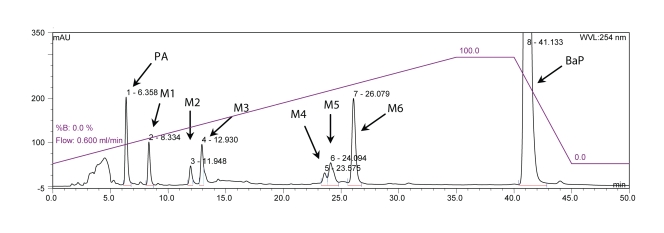
HPLC separation of the BaP metabolites generated by rat hepatic microsomes using a Nucleosil^®^ C18 reverse phase column. A linear gradient elution from 50% methanol to 85% methanol in 50 min. Flow rate 0.6 ml/min, UV detection at 254 nm. PA, phenacetine, M1-M6, BaP metabolites [BaP-9,10-dihydrodiol (M1), BaP-4,5-dihydrodiol (M2), BaP-7,8-dihydrodiol (M3), one of the BaP diones (1,6 or 3,6 or 6,12-BaP-dione, M4), 9-hydroxy BaP (M5) and 3-hydroxy BaP (M6)].

In the Procedure 4 the experimental conditions were as follows: mobile phase A: 50% acetonitrile (50:50 acetonitrile: water, v/v), mobile phase B: 85% acetonitrile (85:15 acetonitrile: water, v/v). Initial elution conditions were 50% acetonitrile with a linear gradient to 85% acetonitrile in 35 min, then an isocratic elution of 85% acetonitrile in 5 min, a linear gradient from 85% acetonitrile to 50% acetonitrile in 5 min, followed by an isocratic elution of 50% acetonitrile in 5 min. Total run time was 50 min. BaP metabolite separation was performed on a Nucleosil^®^ C18 reverse phase column, (250 × 4 mm, 5 µm; Macherey Nagel). The used gradient program is shown in Table 1. The BaP metabolite peaks (Figure 4) were collected and analyzed by mass spectrometry.

**Table 1:** HPLC conditions used for a step gradient elution of BaP metabolites on a Nucleosil^®^ C18 reverse phase column

Time [min]	Mobile phase A 50% acetonitrile	Mobile Phase B 85% acetonitrile	Flow rate
0	100%	0%	
35	0%	100%	
40	0%	100%	0.6 ml/min
45	100%	0%	
50	100%	0%	

### Mass spectrometry

Mass spectra were measured on a matrix-assisted laser desorption/ionisation reflectron time-of-flight MALDITOF mass spectrometer ultraFLEX (Bruker-Daltonics, Bremen, Germany). Positive spectra were calibrated externally using the monoisotopic [M + H]+ ion of bradykinin 757.399 m/z and CCA matrix peaks 190.050, 379.092 m/z. A 10 mg/ml solution of α-cyano-4-hydroxy-cinnamic acid or 2,5-dihydrobenzoic acid in 50% acetonitrile/0.3% acetic acid was used as a MALDI matrix. A 0.5 µl of sample dissolved in acetonitrile was premixed with a 0.5 µl of the matrix solution on the target and allowed to dry at ambient temperature. The MALDI-TOF positive spectra were collected in reflectron mode.

## Results and discussion

In order to receive the BaP metabolites, which are formed by a combination of CYP- and epoxide hydrolase-mediated reactions, rat hepatic microsomes used in the experiments. Because CYP1A1 is one of the most efficient enzymes metabolizing BaP *in vitro*, hepatic microsomes of rats treated with a CYP1A1 inducer, Sudan I (Lubet *et al*., 1983; Martínek and Stiborová, 2002; Stiborová *et al*., 2002), were employed. Using such microsomes, formation of a spectrum of mono- and di-hydroxylated BaP metabolites as well as quinones (diones) of BaP that were found to be formed by CYP1A1 with epoxide hydrolase (Baird *et al*., 2005; Luch and Baird 2005) was expected.

To improve the existing HPLC chromatographic method analyzing BaP metabolites and to make them sensitive enough to be detected with UV detection, we investigated various elution systems and chromatographic procedures. In addition, two different HPLC analytical columns were examined to evaluate their efficiencies to separate BaP metabolites. The columns Ultrasphere^®^ and Nucleosil^®^ obtained from Beckman-Coulter and Macherey-Nagel, respectively, were utilized for such a study. Constant flow rate of 0.6 ml/min under 35°C was used. To detect BaP and its metabolites, a spectrum of different wavelengths were tested to detect BaP (data not shown). The highest sensitivity to detect BaP and its metabolites separated by HPLC was at 254 nm. Four procedures (Procedures 1, 2, 3 and 4, see the Materials and Methods section) were tested in this work.

Because several methods described in the former studies used methanol/water or acetonitrile/water as mobile phases in HPLC for separation of BaP metabolites, we tested both these mobile phases in different arrangements. First we tried methanol/water as mobile phase with gradient program (30% to 70% methanol), which was analogous to mobile phase used previously by Selkirk *et al*., 1974, but even after optimization and using two chromatographic columns (Nucleosil^®^ C18 and Ultrasphere^®^ reverse phase columns), we were unable to detect any BaP metabolites or even BaP itself under these conditions (see Figure 2 showing the results found using the Procedures 1 and 2). Chromatographic profiles were as almost baseline without elution of BaP and metabolites (Figure 2), probably by retaining the BaP and its metabolites on the resin under such conditions.

Since the methanol/water mobile phase was found to be inappropriate for elution of BaP and its metabolites, we utilized acetonitrile/water as mobile phase in further studies. In this case, we first tried 85% acetonitrile in water (v/v) with an isocratic elution using the Nucleosil^®^ C18 reverse phase column (Procedure 3), however, even after optimization, we did not achieve ideal separation of BaP metabolites formed by rat hepatic microsomes (Figure 3). BaP metabolites eluted between 4 to 11 min, while BaP at 18.6 min (Figure 3). When we changed the elution conditions with acetonitrile/water as mobile phase to the gradient program shown in Table 1 (Procedure 4), six BaP metabolites were almost perfectly separated using the same chromatographic column (a Nucleosil^®^ C18 reverse phase column, Figure 4). The BaP metabolites formed by rat hepatic microsomes, assigned in Figure 4 as metabolites M1-M6 and eluted at retention times of 8.3 (M1), 11.9 (M2), 12.9 (M3), 23.6 (M4), 24.1 (M5) and 26.1 min (M6), were characterized by mass spectrometry and tentatively identified to be BaP-9,10-dihydrodiol (M1), BaP-4,5-dihydrodiol (M2), BaP-7,8-dihydrodiol (M3), one of the BaP diones (1,6 or 3,6 or 6,12-BaP-dione, M4), 9-hydroxy BaP (M5) and 3-hydroxy BaP (M6). Parental BaP was eluted at 41 min. The study resolving which of BaP diones is a metabolite M4 is under way in our laboratory.

## Conclusion

The new HPLC method developed in this work allows for separation of BaP metabolites with increased resolution, simple procedure and high detection sensitivity. Because of controversial results suggesting a more important role of CYP1A1 *in vivo* in BaP detoxification than in its activation (Uno *et al*., 2004; 2006; Arlt *et al*., 2008), BaP metabolism and DNA adducts formation should be re-investigated in more details. The developed HPLC method will be useful for such additional studies, to further define the real roles of the CYP1A1 enzyme both *in vitro* and *in vivo* in activation and detoxification of BaP.
